# Running demands and activity profile of men’s rugby sevens: a tournament scenario

**DOI:** 10.5114/biolsport.2022.107023

**Published:** 2021-07-15

**Authors:** Marcus Lee, Jacky Soo, Vincent Yeo, Abdul Rashid Aziz, Mohammed Ihsan

**Affiliations:** 1 Sport Physiology, Sport Science, National Youth Sports Institute, Republic of Singapore; 2 Sport Physiology, Sport Science and Sport Medicine, Singapore Sport Institute, Singapore, Republic of Singapore; 3 Murdoch Applied Sports Science Laboratory, Murdoch University, Perth, Western Australia; 4 Catapult Sports, Victoria, Australia; 5 Research and Scientific Support, Aspetar Orthopedic and Sports Medicine Hospital, Doha, Qatar; 6 Human Potential Translational Research Program, Yong Loo Lin School of Medicine, National University of Singapore

**Keywords:** Rugby Sevens, Team sports, Match demand, Collision, Tournament, GPS

## Abstract

This study profiled the changes in running performances and collisions within a Rugby sevens tournament. Sixteen male players were equipped with global positioning system units while competing at the 2015 and 2016 Asia Rugby Sevens series held in Colombo and Hong Kong, respectively. Both tournaments consisted of 4 matches each, and were played over 2 days (i.e., 2 matches/day). Total distance (TD) covered increased in match 3 compared with matches 1 (19 ± 19%; p < 0.001) and 2 (16 ± 11%; p = 0.001), whilst a decrease in TD in match 4 compared with match 3 (8 ± 9%; p = 0.019) was observed. Distances covered within 6.1–12 km·h^-1^ and 12.1–14 km·h^-1^ speed bands were generally higher in matches 3 and/or 4 when compared with match 1 and/or 2 (p < 0.05). Frequency of entries into 14.1–18 km·h^-1^ speed zone was decreased in match 4 compared with match 3 (45 ± 41%; p = 0.009), whilst incidences of heavy, very heavy and severe collisions were generally higher in matches 3 or 4 compared with matches 1 or 2 (p < 0.05). In conclusion, while some decrements in the final match were evident, running performance were generally maintained throughout despite the competitive and congested nature of Rugby Sevens tournaments

## INTRODUCTION

Rugby Sevens is a seven-a-side variant of the 15-a-side Rugby Union. Since the inclusion of this sport in the 2016 Rio Olympic Games, there has been considerable increase in the games’ popularity and global participation, and resultantly, an increase in research examining playing demands and athlete profiles [1–7]. Although Sevens is played under similar rules and pitch size compared to the 15-a-side code [3, 5], the number of competing players, match and tournaments durations considerably differ. For instance, competing teams play in two 7-minute halves interspersed with a 2-minute break instead of the usual 40-minute half with 10-minute break that Rugby Union adheres to. Due to the reduced number of players competing in a similar sized pitch, and the shorter duration of each match (i.e., ~14 min), the overall relative running demands, and proportion of high running velocities have been reported to be higher in Sevens (81–123 m·min^-1^ and ~17%, respectively) compared with the 15-a-side code (56–81 m·min^-1^ and 5–12%, respectively) [1–3]. Moreover, profound metabolic alterations including decreases in blood pH (~1.6%) and HCO_3_^-^ (~44%), in line with increases in blood lactate (~380%) concentrations have been reported following a competitive Sevens match [8], highlighting the intense nature of Sevens match-play.

Sevens tournaments are fairly congested, involving 2–3 matches a day over 2 days, with 2–4 h recovery between matches. Such requirement to repeatedly compete within short periods likely results in incomplete recovery and accumulated fatigue. Moreover, non-running physical demands such as collisions (rucks, tackles, mauls, and scrummages etc.) have been shown to impair upper body neuromuscular function, and substantially contribute to the extent of muscle damage and soreness, prolonging recuperation and recovery [9, 10]. Accordingly, a 500% increase in blood creatine kinase concentration has been observed following a Sevens tournament [11]. This increase in blood creatine kinase paralleled a 26% decrease in counter-movement jump performance up to 12 h following a Sevens tournament (5 matches in 2 days), which persisted to be 7% lower compared with baseline values following 60 h of recovery [11].

Research in Rugby Sevens is accumulating, and currently include studies examining the overall and within match running demands [3, 5, 12, 13], positional differences and incidences of collisions [4, 14], as well as physiological recovery following tournaments [11]. However, unlike other team-sport where successive matches are played following short recovery periods [15, 16], there is a dearth specifically examining the changes in activity profile within a Sevens tournament. Indeed, progressive decrements in physical performance during tournaments have been observed in other team-sports such as Rugby League [17], Basketball [18] and Field Hockey [19]. Understanding the potential changes in running and collision activity throughout a tournament is essential for the management of player fatigue and recovery, likely resulting in preserved physical performance and reduced risk of injuries [20]. The time-course changes in a tournament setting may provide specific insights in the context of repeated back to back matches and consecutive match-days, leading to the optimization of physical conditioning and rotation strategies. As such, the purpose of this study is to profile the time-course changes of running demands and physical collisions across matches within a Rugby Sevens tournament.

## MATERIALS AND METHODS

### Participants

Sixteen male Rugby Seven players (age; 25.8 ± 3.8 years, stature; 177 ± 5 cm, body mass; 80.2 ± 7.0 kg, estimated maximal oxygen uptake; 46.7 ± 3.7 ml·kg·min^-1^, 40 m sprint time; 5.40 ± 0.19 s) from the Singapore Rugby Union were assessed during the 2015 and 2016 Asia Rugby 7s Series held in Colombo and Hong Kong, respectively. Selected participants have been playing for the national side for 5.3 ± 4.4 years, and their weekly training volume was 6–10 hours, including 2–3 hours of physical conditioning, and 4–7 hours of skills/tactical training. All players were informed of the requirements of the study, and a written informed consent was obtained prior to data collection. This study was approved by the research ethics committee of the Singapore Sports Institute.

### GPS data acquisition and analysis

Time-motion analyses using global positioning systems (GPS) was undertaken during two tournaments as part of the 2015 and 2016 Asia Rugby 7s Series, which were held in Colombo and Hong Kong, respectively. Both tournaments consisted of 4 matches each, and were played over 2 days. Mean recovery time between matches were 204 ± 106 minutes. All matches consisted of two 7 minute halves, and were played on a full-sized outdoor pitch. Environmental conditions during the tournaments were between 30.2 ± 4.1˚C and 76 ± 16% relative humidity. During each match, players were instrumented (positioned in between the scapula) with a 15 Hz GPS unit (SPI Pro X, GPSports systems, Canberra, Australia) including a built-in tri-axial accelerometer (100 Hz). The 15 Hz GPS units have been shown to demonstrate good reliability indices (typical error of measurement; TEM) for TD (TEM = 1.9%) and distances covered at < 14 km·h^-1^ (TEM = 2.0%), but decreased reliability for distances covered between 14–19.99 km·h^-1^ (TEM = 7.6%) and > 20 km·h^-1^ (12.1%) [21].

Each player wore the same unit for all the matches, turned on 15-min prior to data collection to allow for the acquisition of a stable satellite signal. All GPS-acquired data were downloaded to a personal laptop and analyzed using the manufacturer’s software. GPS data were edited to include exclusively, time spent on the field of play based on substitution times manually recorded during the game. There were on average 4.4 ± 0.5 substitutions per match. With 6, 23 and 69% of all substitutions made in the first-half, at half-time and during the second-half, respectively. Moreover, mean substitution times for the first and second-halves were 145 ± 35 s and 179 ± 93 s, respectively. Edited data were then combined for players who substituted each other, creating an activity profile for each playing position. This approach reflects a specific field position’s work-rate and activity pattern which informs the highest demand for each position [22, 23]. The variables selected for investigation derived from the GPS data were based on previous studies investigating description of match characteristics in men’s Rugby sevens [3–5] and included total distance covered (TD), distances covered in six separate speed zones (0–6 km·h^-1^, 6.1–12 km·h^-1^, 12.1–14 km·h^-1^, 14.1–18 km·h^-1^, 18.1–20 km·h^-1^ and > 20 km·h^-1^), as well as the frequencies of high-speed running bouts (i.e., 14.1–18 km·h^-1^, 18.1–20 km·h^-1^ and > 20 km·h^-1^). Number of moderate accelerations (2 to 4 m·s^-2^) and decelerations (-4 to – 2 m·s^-2^), as well as high accelerations (> 4 m·s^-2^) and decelerations (< -4 m·s^-2^) were also determined [5]. Impacts/collisions were categorized as heavy (7–8 *g*), very heavy (8–10 *g*) or severe (> 10 *g*) [4].

### Statistics

Changes in GPS-derived measures across matches 1–4 were analyzed using linear mixed-model, with matches (i.e., Match 1, Match 2, Match 3 and Match 4) modelled as fixed effect, and players fitted as random effect to account for repeated measurement between matches. Linear mixed model accounts for the unbalanced design as well as missing data from players since players differed in the number of matches participated [24]. Where significant main effects (p < 0.05) were evident, Bonferroni analysis was used for post-hoc comparisons. Effect size (ES) with 95% confidence limit (CL) were calculated by dividing the mean difference of the change score with the standard deviation (SD), with SD obtained by square rooting and summing the variances from the mixed linear model [25]:


$$\mathrm{ES}=\mathrm{Differences}/\left(\sqrt{\mathrm{Match}\;\mathrm{variance}}+\sqrt{\mathrm{Residual}\;\mathrm{variance}}\right)$$

The following thresholds were used: small (≥ 0.2), moderate (≥ 0.6), large (≥ 1.2) and very large (≥ 2.0) [26]. All data are presented as mean ± SD. All statistical analyses were performed using the SPSS statistical software V.24.0 (IBM Corp., Armonk, NY, USA). The significance level was set at p < 0.05.

## RESULTS

Overall running distances (i.e., pooled data from both tournaments, n = 56 files;) for TD, as well as for the 6 speed zones (i.e., 0–6 km·h^-1^ to > 20 km·h^-1^) were 1385 ± 150 m (TD), 470 ± 59 m (0–6 km·h^-1^), 351 ± 96 m (6.1–12 km·h^-1^), 126 ± 32 m (12.1–14 km·h^-1^), 206 ± 52 m (14.1–18 km·h^-1^) 75 ± 25 m (18.1–20 km·h^-1^) and 156 ± 68 m (> 20 km·h^-1^). Mean collisions were 23 ± 13, 16 ± 10 and 8 ± 3, for heavy (7–8 *g*), very heavy (8–10 *g*) and severe (> 10 *g*) classifications, respectively. Moderate and high accelerations amounted to 19.5 ± 4.9 and 0.6 ± 0.7 efforts, whilst moderate and high decelerations amounted to 20.0 ± 5.4 and 3.6 ± 1.9, respectively.

Significant main effects for matches were noted for changes in TD (p = 0.001), as well as distance covered in 0–6 km·h^-1^ (p = 0.036), 6.1–12 km·h^-1^ (p < 0.001) and 12.1–14 km·h^-1^ (p = 0.004) speed bands (Table 1).

**TABLE 1:** Total distance covered and distance covered at 6 speed zones ranging from 0 to > 20 km·h-1

Variable	Match 1 (m)	Match 2 (m)	Match 3 (m)	Match 4 (m)	p-value
**Total distance**	1305 ± 181	1326 ± 87	1531 ± 92^a,b^	1398 ± 114^c^	0.001
**0–6 km·h^-1^**	478 ± 49	437 ± 58	482 ± 45	487 ± 73	0.036
**6.1–12 km·h^-1^**	298 ± 62	298 ± 55	439 ± 103^a,b^	387 ± 87^a,b^	< 0.001
**12.1–14 km·h^-1^**	109 ± 31	118 ± 21	152 ± 34^a,b^	129 ± 26	0.004
**14.1–18 km·h^-1^**	183 ± 65	204 ± 35	235 ± 49	205 ± 44	0.115
**18.1–20 km·h^-1^**	78 ± 23	83 ± 28	74 ± 24	64 ± 22	0.073
**> 20 km·h^-1^**	157 ± 71	186 ± 63	150 ± 63	128 ± 67	0.140

Data are presented as mean ± SD.

a, b, c denotes significant difference (p < 0.05) from match 1, match 2 and match 3, respectively.

Specifically, TD in match 3 was higher compared with matches 1 (19 ± 19%; p < 0.001; ES = 1.51 [95% CL: 0.86, 2.15]) and 2 (16 ± 11%; p = 0.001; ES = 2.31 [1.49, 3.13]), whilst a decrease in TD in match 4 was observed compared with match 3 (8 ± 9%; p = 0.019; ES = 1.05 [0.41, 1.69]). Within 6.1–12 km·h^-1^, running distances were significantly higher in matches 3 and 4, compared with 1 and 2 (p < 0.05; ES = 1.43–2.34). In 12.1–14 km·h^-1^, running distance in match 3 was significantly higher compared with match 1 (50 ± 42%; p = 0.005; ES = 1.37 [0.64, 2.10]) and match 2 (31 ± 32%; p = 0.011, ES = 1.50 [0.64, 2.35]).

**FIG. 1:**
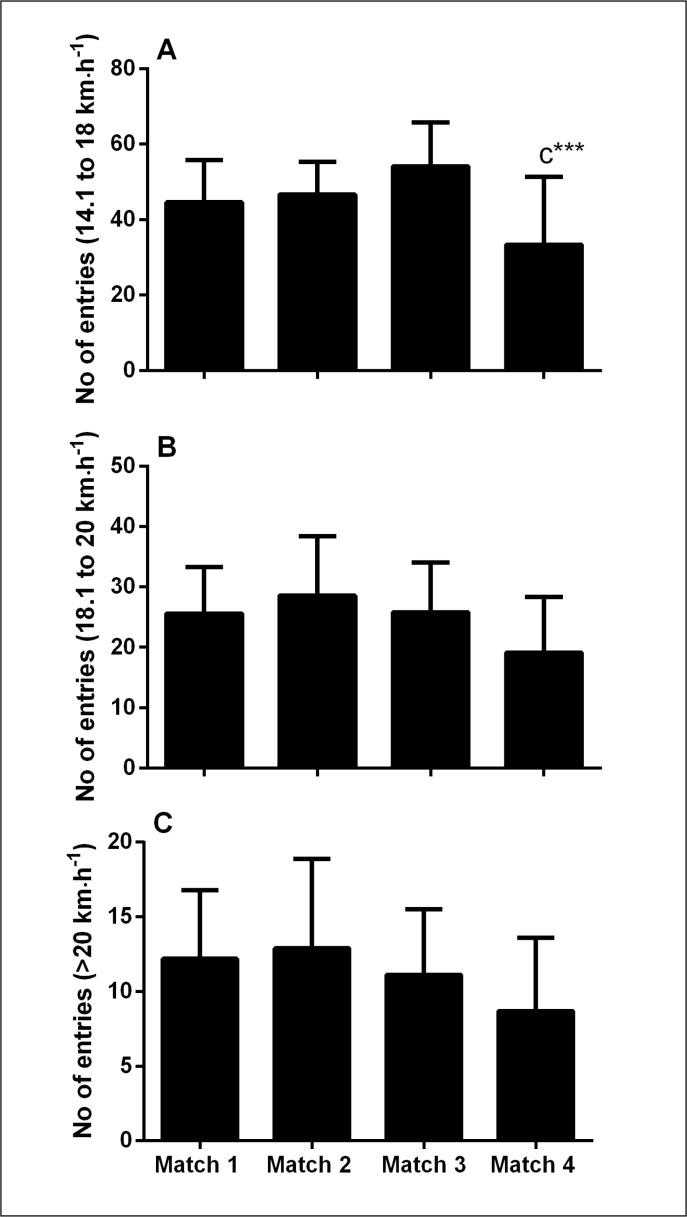
Number of entries into 14–18 km·h^-1^ (panel A), 18–20 km·h^-1^ (panel B) and > 20 km·h^-1^ (panel C) speed bands. ^c^significantly different from match 3 (p < 0.05). ***Large effect size.

Significant main effects for matches were observed on the frequency of 14.1–18 km·h^-1^ running bouts (Figure 1, p = 0.012), where frequency in match 4 was decreased compared with match 3 (45 ± 41%; p = 0.009; ES = 1.89 [0.82, 2.96]). No effects were observed for running bouts with speed ranges of 18.1–20 km·h^-1^ (p = 0.066) and > 20 km·h^-1^ (p = 0.155).

**FIG. 2:**
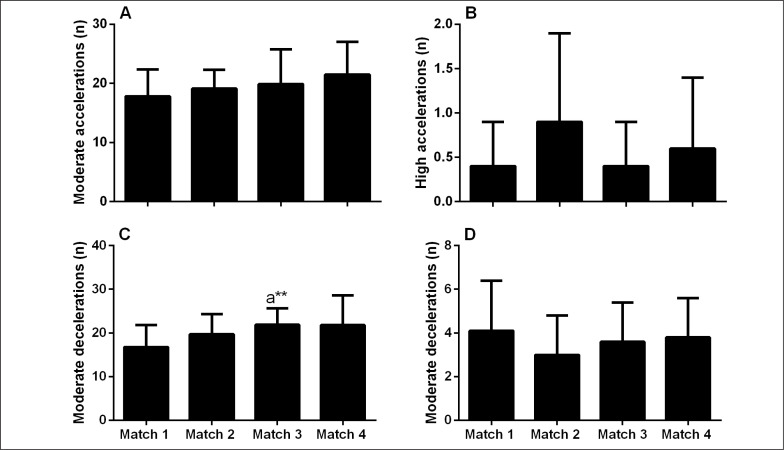
Number of moderate (2 to 4 m·s-2, panel A) and high (> 4 m·s^-2^, panel B) accelerations, as well as moderate (-4 to 2 m·s^-2^, panel C) and high decelerations (< -4 m·s^-2^, panel D). ^a^significantly different from match 1 (p < 0.05). **Moderate effect size.

Significant main effects for matches for the changes in moderate decelerations were observed (Figure 2C, p = 0.02), where increased number of decelerations were observed in match 3 compared with match 1 (41 ± 46%; p = 0.02; ES = 1.05 [0.39, 1.70]).

**FIG. 3:**
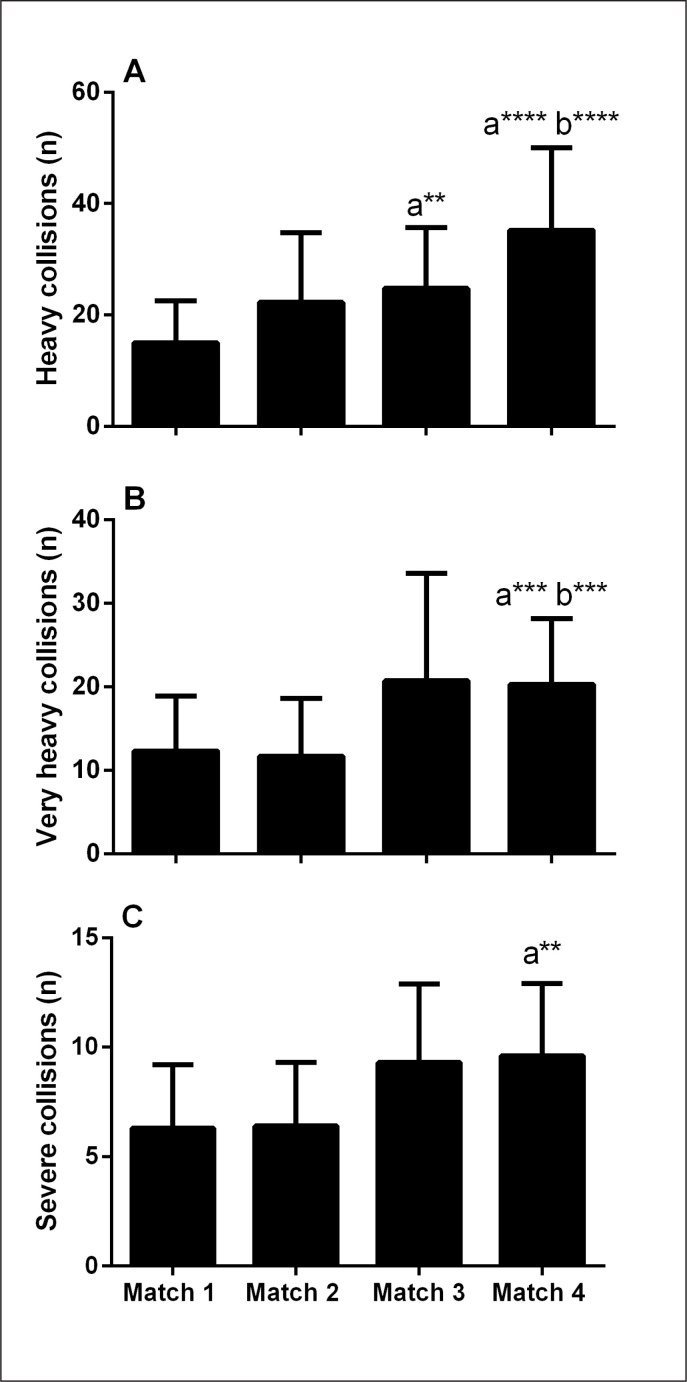
Number of heavy (7–8 g, panel A), very heavy (8–10 g, panel B) and severe (> 10 g, panel C) collisions. ^a and b^significantly different from match 1 and 2, respectively (p < 0.05). **Moderate effect size, ***Large effect size, ****Very large effect size.

Significant main effects for matches were observed for heavy (p = 0.001), very heavy (p = 0.004) and severe (p = 0.01) collision (Figure 3). Specifically, heavy collisions (7–8 *g*) increased in matches 3 (99 ± 139%) and 4 (213 ± 204%), compared with match 1 (p ≤ 0.05; ES = 1.36–2.81), and in match 4 compared with match 2 (100 ± 83%; p = 0.009; ES = 2.14 [0.96, 3.32]). Very heavy collisions were higher in match 4 compared with matches 1 (126 ± 138%; p = 0.027; ES = 1.28 [0.45, 2.11]) and 2 (208 ± 285%; p = 0.017; ES = 1.32 [0.51, 2.13]). Lastly, severe collisions were higher in match 4 compared with match 1 (101 ± 132%; p = 0.046; ES = 1.19 [0.34, 2.04]).

## DISCUSSION

This study provides novel data identifying the changes in movement activity across multiple fixtures within a Sevens tournament. Given that Sevens is played at a substantially greater intensity compared with other Rugby codes [5, 8], and contested in a tournament fashion where 4–6 matches are played over 2–3 days, it is tenable to assume that a profound decline in physical performance may be observed as the tournament progressed. In contrast, the main findings showed that running performances were maintained or even improved in the first 3 matches of the tournament. However, a decrease in TD was observed in match 4, which coincided with decreased frequencies of moderate speed (14–18 km·h^-1^) running bouts, as well as increased incidence of collision events. As such, the present study demonstrates that while some decrements in the final match were evident, running performances were generally maintained throughout despite the competitive and congested nature of Rugby Sevens tournaments.

To date, research examining Sevens match-play has been limited to identifying movement activities within a game [3, 13], between halves [5], across playing positions [4], and between differing playing levels [5]. However, there is limited information, regarding the changes in movement activity across multiple fixtures within a Sevens tournament, although similar research has been conducted in other team-sports such as Field Hockey [15, 27], junior Rugby League [28], Basketball [18] and Soccer [29]. In this regard, the current study provides novel data in identifying the changes in movement patterns within a Rugby Sevens tournament. Our findings are consistent with previous studies in junior Rugby League players, where running performances were generally maintained over the initial periods of the tournament, whilst a slight decrease in performance was observed in the latter stages [17, 28]. In contrast, others have demonstrated a progressive decline in match running performance over the course of a tournament in elite Field Hockey, Basketball and Soccer [18, 19, 29]. The reasons underpinning such distinct findings are unclear, although player fitness (relative to the level of play) has been suggested as an important determinant regulating match intensity across intensified tournament periods [28]. Pacing mechanisms could also explain the current findings, where the distribution of efforts throughout the tournament are regulated and optimized based on prior knowledge such as total matches anticipated and level of opponents [30]. While this model may explain to some extent the regulation of effort within matches, it remains to be verified if such mechanisms can be extended to explain the regulation of effort within tournaments.

Whilst physical performances were mostly maintained across the tournament, a decrease in TD was observed in match 4 compared with match 3 (Table 1). It is worth noting that the decrease in TD was largely due to the reduced distance covered at lower running speeds (6.1–14 km·h^-1^, Table 1), with no observed changes in higher intensity activity (i.e., higher running speeds, or acceleration and deceleration events, Table 1, Figures 1 and 2). The current observations are in contrast to findings in elite Australian Football where the fatigue during match-play was characterized by an increase in slower running speeds and reduced acceleration events [31]. Our findings are more consistent with a pacing paradigm, where fatigue experienced by players may be mitigated by reducing lower-intensity running, such that resources are available for high-intensity activities, presumably more associated with important moments of the match [32, 33]. Unfortunately, neither accelerometer load (contribution of 3 vectors etc.), nor post-match measures of neuromuscular function were examined in association with the current (tournament) activity profile. Alternatively, the decrease in TD could be simply due to the observed increase in collision events (Figure 3), as an increase in the number of contacts has been shown to decrease running activity in rugby small-sided games [34]. Taken together, a combination of the above factors, in addition to match context such as scoreline, opposition level likely influences match activity profile, and therefore need to be considered when interpreting the running activity profiles.

The authors are aware of some studies that have quantified collision demands in Rugby Sevens [4, 14]. The number of impacts (> 7 *g*) reported is comparable to the current observation (i.e, mean > 40 impacts). This study has nevertheless extended these findings by profiling the changes in collision, demonstrating increased impacts in the latter (i.e., 3 and 4) compared with the initial matches (i.e., 1 and 2) of the tournament (Figure 3). Contextual factors such as tournament stage, match importance and tactics likely account for such findings. Accumulated fatigue may have also resulted in the reduced ability of the players to successfully evade contacts. The development of physical fatigue, presumably more pronounced at the latter stages of the tournament, coupled with increased incidences of collision events present a substantial injury risk, given that physical collisions account for 78% of all injuries during Sevens match-play [35].

The following limitations must be considered when applying the present study’s findings. Objective measures of performance recovery (e.g., neuromuscular assessments) were not examined in the current study, which limits identification of players’ physiological state across the tournament period. Inclusion of objective measures of performance recovery alongside collision data is warranted in future studies to determine plausible associations and countermeasures. Nevertheless, it is acknowledged that collision technologies in GPS wearables are generally developed for the Fifteens game, and may require fine-tuning for increased validity and reliability in Sevens [4, 36]. Lastly, movement patterns and match activities were profiled only for the Singapore Rugby team in this study. It is acknowledged that contextual factors such as opponent level, match status and tactics amongst others may influence running performance and collision [37, 38], which might limit generalization of the findings. Future studies should therefore consider analyzing match characteristics of all teams during the tournament, taking into account contextual factors (e.g., match results), which would allow for a more complete understanding of the activity profiles of Rugby Sevens.

## CONCLUSIONS

This study examined the changes in match activity within a Rugby Sevens tournament. Despite the intense physical demands inherent within the Sevens game, the current findings show that running performances were maintained for most of the tournament (i.e., match 1 to 3). However, a decrease in TD was observed in match 4, coinciding with decreased frequencies of moderate (14–18 km·h^-1^) running bouts, as well as increased incidence of collision events. Despite physical performances being mostly preserved, the authors do not discount the possibility of accumulation of physical fatigue in line with the progression of the tournament. The increased incidence of collision events at the latter, presumably more fatigued periods of the tournament present considerable risks for injury. Wellness or recovery monitoring tools are therefore important to identify players who may be particularly fatigued, and should be utilized towards player rotation/substitution strategies to minimize fatigue/injury and maintain the team’s work rate. Rugby Sevens training should incorporate collision-related (impact, evasive and defensive) drills in a fatigued state to develop tolerance and movement strategies mitigating collision loads. Consequently, players’ response to this training load should be carefully managed and structured within the team’s meso and micro-cycle in relation to the tournament phase.
